# Mutations of *METTL3* predict response to neoadjuvant chemotherapy in muscle-invasive bladder cancer

**Published:** 2021-06-05

**Authors:** Zhao Yang, Zongyi Shen, Di Jin, Nan Zhang, Yue Wang, Wanjun Lei, Zhiming Zhang, Haige Chen, Faiza Naz, Lida Xu, Lei Wang, Shihui Wang, Xin Su, Changyuan Yu, Chong Li

**Affiliations:** ^1^College of Life Science and Technology, Beijing University of Chemical Technology, Beijing 100029, China; ^2^College of Life Science, Key Laboratory of Protection and Utilization of Biological Resources in Tarim Basin of Xinjiang Production and Construction Corps, Tarim University, Alar 843300, Xinjiang, China; ^3^Department of Urology, Renji Hospital, School of Medicine, Shanghai Jiao Tong University, Shanghai 200127, China; ^4^Department of Cancer Research, Novogene Bioinformatics Institute, Beijing 100016, China; ^5^Core Facility for Protein Research, Institute of Biophysics, Chinese Academy of Sciences, Beijing 100101, China; ^6^Zhongke Jianlan Medical Research Institute, Beijing 101400, China

**Keywords:** muscle-invasive bladder cancer, neoadjuvant chemotherapy, METTL3, pathological response, biomarker

## Abstract

**Background and Aim:**

Neoadjuvant chemotherapy (NAC) followed by radical cystectomy is the current gold standard treatment for muscle-invasive urothelial bladder cancer (MIBC). Nonetheless, some MIBC patients showed limited pathological response after NAC. Herein, we used whole-exome sequencing (WES) to identify genetic mutations in MIBC that can predict NAC response.

**Methods:**

Forty MIBC patients were enrolled in this study, in which 33 were successfully examined by WES and Sanger sequencing in the discovery cohort (*n*=13) and the validation cohort (*n*=20), respectively. ANNOVAR software was used to identify the potential mutations based on the data of WES. In addition, tumor-specific somatic mutations including single nucleotide variants and indels were called with the muTECT and Strelka software. The mutational analysis of specific genes was carried out based on the data from cBioPortal for Cancer Genomics.

**Results:**

In the discovery cohort, the mutation frequencies of *TP53*, *MED16*, *DRC7*, *CEND1*, *ATAD5*, *SETD8*, and *PIK3CA* were significantly higher in 13 MIBC patients. Specifically, the presence of somatic mutations of *APC*, *ATM*, *CDH9*, *CTNNB1*, *METTL3*, *NBEAL1*, *PTPRH*, *RNASEL*, and *FBXW7* in NAC responder signifies that these mutations were potential predictors of pathological response to NAC. Furthermore, somatic mutations of *CCDC141*, *PIK3CA*, *CHD5*, *GPR149*, *MUC20*, *TSC1*, and *USP54* were exclusively identified in NAC nonresponders, suggesting that these mutations may participate in the process of NAC resistance. In the validation cohort, the somatic mutations of *CDH9*, *METTL3*, and *PTPRH* were significantly enriched in NAC responders while the somatic mutation of *CCDC141* was significantly enriched in NAC nonresponders. Furthermore, survival analysis revealed that the patients expressing mutated *METTL3* have a longer overall survival and disease- or progression-free survival than the patients acquiring wild-type *METTL3*.

**Conclusion:**

The somatic mutation of *METTL3* can be a potential predictive biomarker of NAC response in MIBC patients.

**Relevance for Patients:**

MIBC patients bearing mutated *METTL3* display a pathological response to NAC and have a significantly longer overall survival or disease/progression-free survival as compared to the patients bearing wild-type *METTL3*. Thus, the somatic mutation of *METTL3* is a potential biomarker for predicting response to NAC in MIBC patients, assisting doctors in making the clinical decision.

## 1. Introduction

Regarded as the fourth most common type of cancer in men worldwide, the incidence of bladder cancer (BC) in men is 4 times higher than in women with approximately 550,000 new cases reported annually [1,2]. Urothelial bladder carcinoma is clinically categorized into two types: Non-muscle-invasive urothelial BC (NMIBC) and muscle-invasive urothelial BC (MIBC). In NMIBC, the cancer cells lie on the superficial surface of the bladder wall. In MIBC, the cancer cells spread into the bladder wall and further metastasize to the other parts or organs [3]. Accounting for about 75% of BC cases, NMIBC patients generally have a favorable overall survival rate but a high recurrence rate [4,5]. Apart from that, MIBC cases account for approximately 25% of all BC cases, and the patients need to be treated with more extensive care and much time is needed for management of the MIBC patients [6]. Compared to NMIBC patient, a MIBC patient has a relatively lower 5-year survival rate and a worse prognosis [7].

To date, the current standard treatment for high-risk MIBC includes cisplatin-based neoadjuvant chemotherapy (NAC), followed by radical cystectomy [8]. Although exhibiting positive therapeutic effects [9,10], the long-term survival rates of MIBC patients receiving this treatment have been remaining unchanged for decades [11]. In addition, the fact that two-thirds of MIBC patients showed partial or no pathological response toward NAC was the reason of delayed surgery and worsened prognosis [12]. Hence, this implies that the pathological response of MIBC patients receiving NAC is strongly associated with survival benefits [13]. Although NAC therapeutic agents were well-tolerated in MIBC patients, the exact toxicity profiles of these therapeutic agents and how it can be adjusted to maximize pathological response without disrupting the healthy cells remained elusive [6]. Therefore, it is imperative to decipher the key players that determine pathological response to NAC in MIBC patients for improving their prognosis.

The emergence of next-generation sequencing (NGS) and comparative bioinformatics analysis has illuminated our understanding of genomic landscape of cancer development and progression. Their application has assisted in the discovery of therapeutic targets as well as the development of targeted therapy and biomarker-based diagnostic tools, providing better solutions for treating recalcitrant cancers [14,15]. Hence, the identification of molecular biomarkers helps predict the pathological response to NAC and provides invaluable information for designing personalized treatment based on the molecular profile of MIBC patients [12,16]. Herein, we identified the biomarkers which can predict the pathological response after NAC treatment in MIBC patients. Through whole-exome sequencing (WES) and mutational studies, we demonstrated that the somatic mutation of *METTL3* is a potential biomarker for predicting response to NAC in BC patients.

## 2. Methods and Materials

### 2.1. Study design and patient selection

In this study, 40 patients were recruited at the Renji Hospital, School of Medicine, Shanghai Jiaotong University from 2016 to 2019. Informed consents were obtained from the patients, and this study was approved by the Research Ethics Board at Shanghai Jiaotong University. The patients who underwent transurethral resection of bladder tumor (TURBT) and were diagnosed with MIBC were selected in this study. The inclusion criteria of MIBC patients include patients with primary carcinoma of the bladder (transitional cell cancer) and clinical stages of T2-4a, N0 or N+, M0 based on American Joint Committee on Cancer (AJCC) guidelines, and whose condition is operable. Besides, BC patients who had complete tumor resection, no evidence of stromal invasion of prostate, adequate renal, hepatic, and hematological functions to tolerate systemic chemotherapy and radical cystectomy were included in this study. In contrast, the patients with distant metastases, unresectable tumor, and other severe diseases, such as heart and renal failure, were excluded in this study.

After DNA sample collections, the patients underwent two cycles of 21-day NAC treatment, which includes 1000 mg/m^2^ gemcitabine over 30-60 min on days 1 and 8, and 70 mg/m^2^ cisplatin on day 2. Following the NAC treatment and surgery, pathological response was assessed by trained physicians. The responders are defined as patients having pathological response (ypT0N0 or ypT1/a/cis) and the nonresponders as those with no response (ypT2+, nonresponders). The patients were divided into discovery and validation cohorts. Each cohort consists of 20 patients. Seven out of 20 patients were excluded from the discovery cohort due to technical failures that happened during DNA extraction, library preparation, and exome sequencing. In the discovery cohort, five patients showed pathological responses while eight patients showed no response. In the validation cohort, 16 patients showed pathological response and four patients showed no response.

### 2.2. Sample collection and preparation

Tumor tissue and peripheral blood specimens were collected from the same patient through TURBT and venepuncture, respectively. Then, tumor tissues and peripheral blood cells were frozen in liquid nitrogen, followed by storage in the ultralow temperature freezer. The genomic DNA of both tumor tissue and peripheral blood samples was extracted using the TIANamp Genomic DNA Kit (TIANGEN, China, DP304) based on the protocols recommended by manufacturer. After DNA extraction, the concentration and purity of DNA were determined using the NanoDrop™ One Microvolume UV-Vis Spectrophotometer (Thermo Scientific, US, ND-ONE-W A30221). The DNA samples were either used for the sequencing studies or stored for future studies.

### 2.3. DNA library preparation for WES in discovery cohort

The extracted DNA samples were used for the DNA library construction and whole-exome enrichment using SureSelect Human All Exon Platform (Agilent Technologies, USA) [17]. First, the genomic DNA was fragmented into the length of 180-280 bp using focused-ultrasonicator (Covaris, USA). The fragmented DNA was purified using Agentcourt AMPure XP reagents (Backman Caulter, USA).

The whole-exome library enrichment was conducted using SureSelect Human All Exon Kit (Agilent Technologies, USA, G3370C) based on manufacturer’s recommended protocols. Briefly, the purified DNA was end-repaired and then adenine-tailed. The indexing-specific paired-end adaptors were ligated to the both ends of DNA to generate a fragment library. After PCR amplification, the fragment library was hybridized with approximately 543,872 biotin-conjugated capture oligos. About 334,378 exons of 20,965 genes were captured with streptavidin-conjugated magnetic beads. The hybridized DNA was PCR amplified using SureSelect Human All Exon Kit. Next, the concentration of amplified fragment library was measured using NanoDrop™ One Microvolume UV-Vis Spectrophotometer (Thermo Scientific, US, ND-ONE-W A30221), and further diluted into 1 ng/μL. The length of the DNA library was confirmed using Agilent 2100 Bioanalyzer coupled with High Sensitivity DNA kit (Agilent Technologies, USA). The optimal amount of final exome libraries was quantitated using quantitative PCR and determined to be >2 nM to ensure the quality of final exome libraries. The final exome libraries sample was sequenced using Illumina Hiseq 2000 platform to generate 2×100 bp.

To validate the result of WES, semi-quantitative PCR was carried out with primers whose sequences are listed in Supplement Table 1. All PCR products were examined by Sanger sequencing and the putative somatic mutations of the discovery cohort were selected according to the reference sequence of peripheral blood specimens from the same patient. The raw data could be given upon request.

**Supplementary Table 1:** PCR primer sequences for selected genes

Gene	Forward primer	Reverse primer	Application
*CCDC141-888*	GTCCTCAGGAGCTAAACTCTAGCA	CATCTCCAGGTAACTAACAATGGC	Sanger
*CCDC141-971*	CTTTGCAGGAGGTGCAGGAAGATA	TACACAAGGAGACAAGGCATTCGG	Sanger
*PIK3CA-076*	AGGAACACTGTCCATTGGCA	GCTGAACCAGTCAAACTCCAACTC	Sanger
*PIK3CA-091*	GATTGGTTCTTTCCTGTCTCTG	TTTAGCACTTACCTGTGACTCC	Sanger
*USP54-383*	TGTGCCCCAAATCAGTGCCTATCT	CTGGATGAATTGCAGGAAGAGG	Sanger
*USP-139*	ACTGGAGAAGCCATGGGCAAATAC	TCCCCTCATGATTCCCATACGTGT	Sanger
*CHD5-426*	ACACACCTATGGTTCAGGATTCGG	TGGGTGAAGGAGCTACAGGTGA	Sanger
*CHD5-655*	AGAAAGAGATGCGGGAACAGACAG	CTGAGGATGAGGATGAGGACTT	Sanger
*GPR149-882*	TTCCTGGTAGTTGGAGTGGAGTCT	GTCCCCGGTTACTTCCAATTTCTG	Sanger
*GPR149-736*	GTTCTGCCTGTGTGCTTCTACTGT	TATGCCCTTGCCATTCCCTTGT	Sanger
*MUC20-843*	GCATCACAGAAATAGAAACAACGACTTCCAG	TCTTTCTGTGGCGCTGTTAGTG	Sanger
*TSC1-693*	CCCGGCCCAAACAAGATCTTTAAC	AAGGCAGAACTGTAATGCT	Sanger
*RNASEL-491*	AGCCTCCACATCACTATCGTCAGA	CCTTTTATCCTCGCAGCGATTG	Sanger
*RNASEL-809*	CGAAGCAGAAGTTCCACAATGTCC	AGCAGGTGGCATTTACCGTCAT	Sanger
*NBEAL1-514*	CCAGTGGCTTCCAGAACTACAATC	AGTTTTCGGGCCATTGTCAGGA	Sanger
*CTNNB1-137*	GGACAAGGAAGCTGCAGAAGCTAT	CTCAAGCCAGGGAAACATCAATGC	Sanger
*CDH9-861*	GGGCAGAGCTTACTAAGCAGTATG	CTCCCCGAGGTCACAAATTCTT	Sanger
*CDH9-395*	GCTTGGTGCGACGTAGCATTTTA	GTTGTGGGAAAGTGAAACTCAAGC	Sanger
*APC-437*	TATGGTCAATACCCAGCCGACCTA	CCCCGTGACCTGTATGGAGAAA	Sanger
*FBXW7-228*	CTAAGGTGGCATTCCTCTTAT	TCATCACACACTGTTCTTCTGGA	Sanger
*METTL3-704*	CTGCTGCTCACCAAGCAGTGTTC	ATGGAGTTGGGGAGAGAATGTCTA	Sanger
*METTL3-651*	ATGGCAGAGAGCTTGGAATGGTCA	GCTGTGTCCATCTGTCTTGCCATCT	Sanger
*PTPRH-222*	CCCTCTGCTCTTCCAGGAATCT	AGATGAGAGAGAGTCGGCCGTTGA	Sanger

### 2.4. Data processing and detection of somatic mutations in MIBC patients

After filtering out the sequence reads containing sequencing adaptors and low-quality reads with more than five unknown bases, the high-quality reads were aligned to the NCBI human reference genome (hg19) using Burrows-Wheeler Aligner (BWA) and Samblaster software. Local realignment of the BWA aligned reads and base quality was assessed using Genome Analysis Toolkit (GATK) (1.2-44-g794f275). ANNOVAR software [18] was used to identify the potential mutations. In this process, the inclusion criteria for sequence reads were applied: (i) Both the tumors and matched peripheral blood specimens should be covered sufficiently (≥10×) at the genomic position being compared; (ii) the average base quality for the specific genomic position should be at least 15 in both tumors and matched peripheral blood specimens; (iii) the variants should be supported by at least 10% of the total reads in the tumors while no high-quality variant-supporting reads are allowed in normal control; and (iv): the variants should be supported by at least five reads in the tumors.

Tumor-specific somatic mutations were detected using the DNA extracted from the matched blood samples of the same patient as reference Germline mutations were identified and filtered by WES. Then, the Germline mutations were effectively removed. Variations including single nucleotide variants (SNVs) and indels in the tumors were called with the muTECT [19] and Strelka [20] software. Somatic mutations that meet the following criteria were excluded from the study: (i) Variants with Phred-like scaled consensus scores or SNP qualities <20; (ii) variants with mapping qualities <30; (iii) indels represented by only one DNA strand; and (iv) substitutions located 30 bp around predicted indels. To filter out the false positive results, such as repeated sequences, simulated reads (80 bp in length) containing the potential mutations were generated and aligned to the reference genome. If more than 10% of the simulated variant-containing reads could not be uniquely mapped to the reference genome, this variant would be eliminated. To eliminate any previously described Germline variants, the somatic mutations were cross-referenced against the dbSNP (version 137). Any mutations presented in the above-mentioned data sets were filtered out and the remaining mutations were subjected to subsequent analyses. In these two processes, MutSigCV_1.4 was used to identify the genes that were significantly mutated in the MIBC patients who responded and do not respond to NAC.

### 2.5. Mutational signature analysis

Mutational signature characterizing the mutational processes in the discovery cohort was identified using steps described elsewhere [21]. In brief, all somatic SNVs detected in the 13 patients were included to calculate the fraction of mutations at each of the 96 mutated trinucleotides. Nonnegative matrix factorization (NMF) was employed to extract biologically meaningful mutational signatures which were displayed by a different profile of the 96 potential trinucleotide mutations. Evaluation of NMF decompositions suggested that the three mutational signatures were superior, given the marginal efficiency of the fourth signature. Furthermore, the relative contributions of the three signatures to each case were estimated.

### 2.6. Sanger sequencing for validation cohort

The DNA of validation cohort was amplified using ProFlex PCR system (Applied Biosystems, US) and the primer sequences are listed in Supplement Table 1. Briefly, PCR products were generated in 30 PCR cycles from a 20-μL reaction mixture containing 30 ng of DNA and 1 U of Platinum Taq polymerase (Life Technologies, US, 18038042). The PCR products were examined by Sanger sequencing using CFX384 TOUCH Real-Time PCR Detection System (Bio-Rad, US).

### 2.7. Comparison of somatic mutations in MIBC patients between multiple independent cohort studies

The results of the mutational analysis of this study were compared with those of other studies. Based on the cBioPortal for Cancer Genomics (https://www.cbioportal.org/), the cohort of Robertson *et al*. [22] was selected for comparison of somatic mutations between NAC responder and nonresponder.

### 2.8. Statistical analysis

The correlation between genetic mutations and response to NAC was analyzed using the Fisher’s exact test. The analysis of genetic mutations was performed with Benjamini-Hochberg method using GraphPad Prism software version 5. Patients’ demographics, tumor characteristics and pathological findings were analyzed using Mann–Whitney *U-*test or Fisher’s exact test. The survival analysis was analyzed in the cBioPortal for Cancer Genomics (https://www.cbioportal.org/). The results were presented in a Kaplan–Meier curve with *P*-value from a log-rank test. A value of *P*<0.05 was regarded as statistically significant.

## 3. Results

### 3.1. Somatic mutational analysis of MIBC patients via exome sequencing

**Figure 1:**
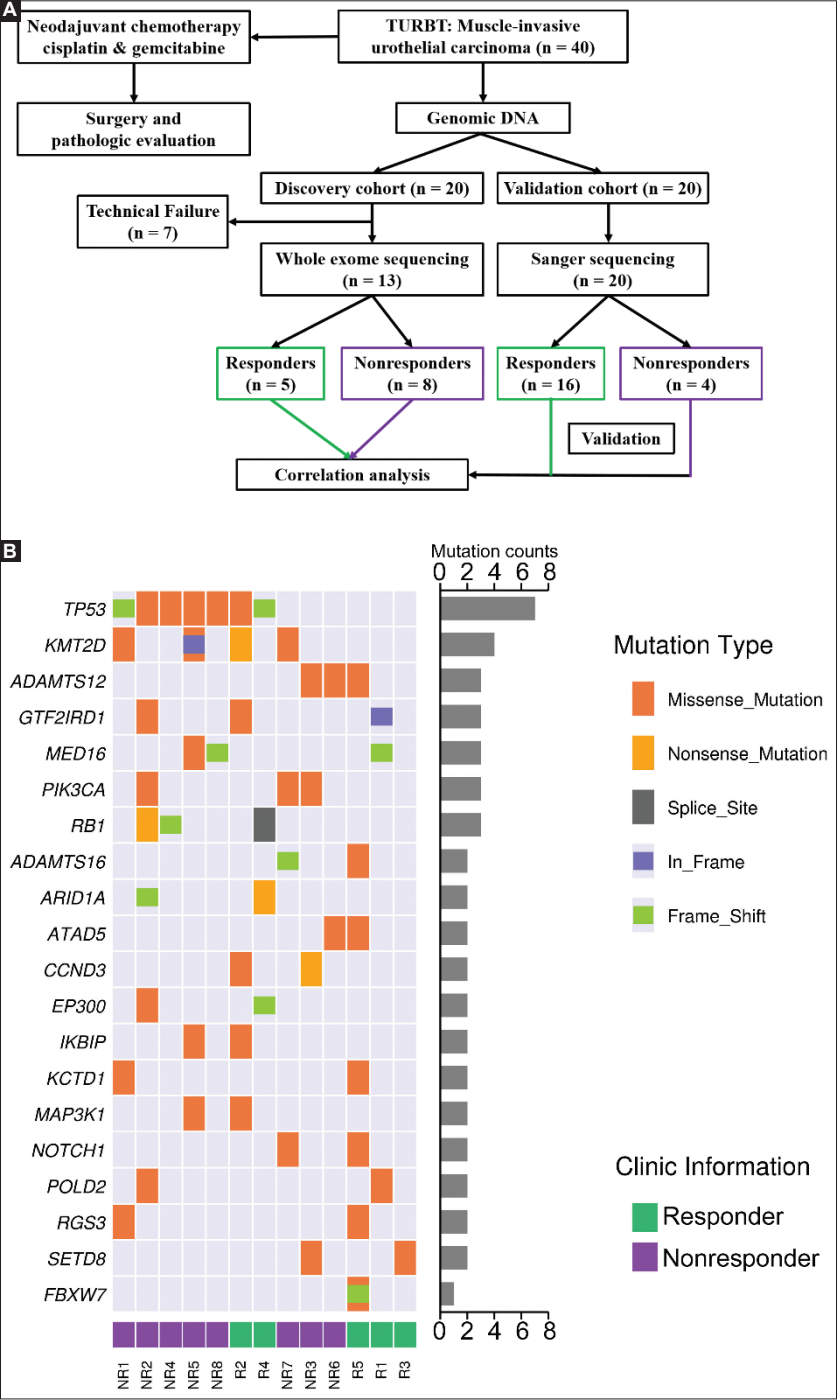
Experimental design and mutation pattern of MIBC patients. (A) Overall workflow of experimental design and patient selection process. The patients were divided into discovery cohort and validation cohort. The somatic mutations were identified through WES and Sanger sequencing that was used in discovery cohort and validation cohort, respectively. The patients were divided into responders and nonresponders based on their pathological response to NAC. In discovery cohort (*n*=13), five patients showed pathological response to NAC (responder) while eight patients showed no pathological response to NAC (nonresponder). In validation cohort (*n*=20), 16 patients showed pathological response to NAC (responder) while four patients showed no pathological response to NAC (nonresponder). TURBT, transurethral resection of bladder tumor. (B) The mutation landscape of the discovery cohort (*n*=13) was displayed. Each column represents a tumor, and each row represents a gene. Genes are listed on the left and the center panel is divided into responders (R, green) and nonresponders (NR, purple). The mutation counts were summarized on the right. *n*, patient number.

To identify the potential biomarkers that predict the response of MIBC patients to NAC, 40 MIBC patients were enrolled in this study. Each patient received 1000 mg/m^2^ gemcitabine over 30–60 min on days 1 and 8, and 70 mg/m^2^ cisplatin on day 2. Treatments were repeated for 21 days with two cycles (Figure 1A and Table 1). After the surgery, the pathological response of the patients was examined by a trained physician following the AJCC guidelines.

**Table 1:** Clinical characteristics of the bladder cancer patients

	Total (33)	Nonresponders (12)		Responders (21)		*P* value
	
		Discovery (8)	Validation (4)	Discovery (5)	Validation (16)	
Female	7	1		6		0.171
Age	60.9	61.1		60.8		0.927
Follow-up (days)	978	964		985		0.906
pT>1	17	9		8		0.019
High Grade	33	12		21		1
Basal Subtype	7	3		4		0.687
pN>0	6	2		4		0.865
pCIS=1	2	1		1		0.679
LVI=1	7	2		5		0.715
OS=1	12	7		5		0.047
*CDH9*	9	0	0	2	7	0.008
*METTL3*	8	0	0	2	6	0.014
*PTPRH*	7	0	0	2	5	0.024
*CCDC141*	5	3	2	0	0	0.013
*PIK3CA*	3	3	0	0	0	0.016
*USP54*	2	2	0	0	0	0.054
*CHD5*	2	2	0	0	0	0.054
*GPR149*	2	2	0	0	0	0.054
*MUC20*	2	2	0	0	0	0.054
*TSC1*	2	2	0	0	0	0.054
*RNASEL*	2	0	0	2	0	0.270
*NBEAL1*	2	0	0	2	0	0.270
*CTNNB1*	2	0	0	2	0	0.270
*APC*	2	0	0	2	0	0.270
*ATM*	2	0	0	2	0	0.270
*FBXW7*	1	0	0	1	0	0.443
*RB1*	3	2	-	1	-	0.830
*FANCC*	1	1	-	0	-	0.410
*FGFR3*	1	1	-	0	-	0.410
*ERBB2*	1	1	-	0	-	0.410
*ERCC2*	2	1	-	1	-	0.720

pT: stage; pN: lymph node metastasis; pCIS: carcinoma *in situ*; LVI: lymph-vascular invasion; OS: overall survival.

The patients were divided into discovery and validation cohorts. Each cohort consists of 20 patients. In discovery cohort, the DNA samples of pre-treatment tumor tissues and peripheral blood specimens from patients were extracted for library preparation and exome sequencing. However, seven out of 20 patients were excluded from this study due to technical failures during the process of DNA extraction, library preparation and exome sequencing. Among 13 patients, five patients showed pathological response (ypT0N0 or ypT1/a/cis, responders) and the remaining eight patients showed no response (ypT2+, nonresponders) (Figure 1A and Table 1). In validation cohort, DNA samples of pre-treatment tumor tissues and peripheral blood specimens from patients were extracted for Sanger sequencing. Among the 20 patients, 16 patients showed pathological response and four patients showed no response (Figure 1A and Table 1).

The clinical characteristics including sex, age, grade, follow-up time, lymph node metastasis (pN), carcinoma *in situ* (pCIS), and lymph-vascular invasion (LVI) showed no significant differences between responders and nonresponders at baseline (Table 1 and Supplementary Table 2). According to TCGA transcriptional subtypes of BC, all samples were divided into luminal subtype (*n*=26) and basal subtype (*n*=7). Neither luminal subtype nor basal subtype was associated with response to NAC (Table 1, *P*=0.687). However, overall survival (OS) and stage (pT) were correlated with nonresponders (Table 1 and Supplementary Table 2).

**Supplementary Table 2:** Clinical characteristics of the bladder carcinoma patients

Patient ID	Patient age (years)	Sex	pT	pN	Grade	pCIS (0, wo carcinoma in situ; 1, carcinoma *in situ*)	LVI (0, wo invasion; 1, v invasion)	pCR (NR, non-response; R, response)	Subtype (L: luminal; B: basal)	Follow-up (days)	Survival (0, Survival; 1, death)
NR1	59	M	T4	0	High	0	0	NR	L	66	1
NR10	59	M	T4	0	High	0	0	NR	L	1095	1
NR11	61	M	T3	0	High	0	0	NR	B	644	0
NR12	62	M	T4	0	High	0	0	NR	B	1424	1
NR2	71	M	Tis	0	High	1	0	NR	B	1180	0
NR3	63	M	T3	2	High	0	1	NR	L	614	1
NR4	66	M	T3	2	High	0	0	NR	L	832	0
NR5	64	M	T4	0	High	0	0	NR	L	1451	1
NR6	50	M	T1	0	High	0	1	NR	L	1857	0
NR7	72	M	T1	0	High	0	0	NR	L	1274	1
NR8	60	M	T3	0	High	0	0	NR	L	743	0
NR9	46	F	T3	0	High	0	0	NR	L	393	1
R1	65	F	T0	0	High	0	0	R	L	1250	0
R10	66	M	T4	2	High	0	1	R	L	479	1
R11	41	M	T1	1	High	0	0	R	L	458	0
R12	71	M	T1	0	High	0	0	R	L	727	0
R13	63	M	T1	0	High	0	0	R	L	1387	0
R14	65	M	T3	3	High	0	1	R	L	1100	1
R15	66	F	T3	0	High	0	1	R	B	174	1
R16	60	M	T2	0	High	0	0	R	B	427	0
R17	57	F	T3	0	High	0	0	R	B	1079	0
R18	72	M	T1	0	High	0	0	R	L	1554	0
R19	53	M	T1	0	High	0	0	R	L	478	0
R2	57	M	T0	0	High	0	0	R	L	1474	0
R20	56	M	T4	0	High	0	0	R	L	1450	1
R21	77	F	T3	0	High	0	1	R	L	683	0
R3	58	M	T0	0	High	0	0	R	L	1773	0
R4	60	F	T0	0	High	0	0	R	L	1733	0
R5	61	M	T0	0	High	0	0	R	L	1299	0
R6	43	F	T3	2	High	0	1	R	L	736	1
R7	60	M	T1	0	High	1	0	R	L	596	0
R8	61	M	T1	0	High	0	0	R	L	661	0
R9	65	M	T1	0	High	0	0	R	B	1177	0

**Supplementary Figure 1:**
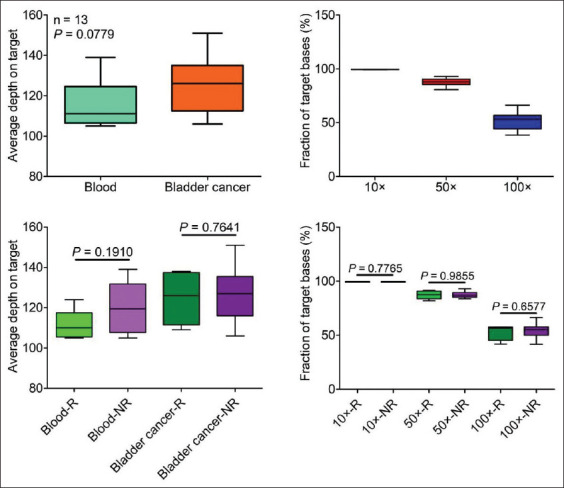
Fold coverage of target region for the peripheral blood and bladder cancer samples from 13 muscle-invasive bladder cancer patients analyzed by whole-exome sequencing. (A) The average depth of of all blood and tumor samples sequenced. (B) The box plot depicts the distribution of fraction of bases covered by at least 10×50× and 100× across the 13 pairs of samples. (C) The box plot depicts the average depth of all blood and tumor samples in responder group (R) and nonresponder group (NR) sequenced. (D) The box plot depicts the distribution of fraction of bases covered by at least 10×, 50× and 100×across R and NR samples.

In exome sequencing, we acquired a mean coverage depth of >100× for all the samples sequenced, with at least 99% of the targeted bases being sufficiently covered (≥10×) (Supplementary Figure 1A and B and Supplementary Table 3). In addition, the average sequencing depth of these two groups remained similar and showed no significant difference (Supplementary Figue 1C and D). After several rigorous bioinformatics analysis steps, up to 4179 somatic mutation candidates and 275 indels were identified in 13 samples (Supplementary Tables 4-6). In total, *TP53*, *MED16*, *DRC7*, *CEND1*, *ATAD5*, *SETD8*, and *PIK3CA* were identified as significantly mutated genes (SMGs, Supplementary Table 7) in the 13 MIBC samples, and 13 key genes associated with the tumorigenesis of BC were illustrated in a heat map (Figure 1B).

**Supplementary Table 3:** Summary statistics of exome sequencing data obtained from the 13 muscle-invasive bladder cancer patients

Sample	NR7-T	NR5-T	NR5-N	NR4-T	R3-N	NR1-N	NR6-N	NR8-T	NR2-N	R1-T
Total	79819762 (100%)	82513204 (100%)	82902250 (100%)	67737844 (100%)	68338360 (100%)	75692246 (100%)	67097014 (100%)	81613844 (100%)	69620894 (100%)	86113090 (100%)
Duplicate	11424259 (14.31%)	12113015 (14.68%)	12381146 (14.93%)	9147266 (13.50%)	11154615 (16.32%)	10554651 (13.94%)	11125654 (16.58%)	12110747 (14.84%)	12755135 (18.32%)	12818834 (14.89%)
Mapped	79768897 (99.94%)	82408159 (99.87%)	82793530 (99.87%)	67605728 (99.80%)	68241168 (99.86%)	75561244 (99.83%)	67034523 (99.91%)	81439252 (99.79%)	69498662 (99.82%)	85981334 (99.85%)
Properly mapped	79478936 (99.57%)	82070966 (99.46%)	82390270 (99.38%)	67278976 (99.32%)	67872162 (99.32%)	75029336 (99.12%)	66595154 (99.25%)	81036584 (99.29%)	69041382 (99.17%)	85619550 (99.43%)
PE mapped	79726766 (99.88%)	82312362 (99.76%)	82702004 (99.76%)	67509836 (99.66%)	68187932 (99.78%)	75447030 (99.68%)	66979242 (99.82%)	81311260 (99.63%)	69429102 (99.72%)	85875438 (99.72%)
SE mapped	84262 (0.11%)	191594 (0.23%)	183052 (0.22%)	191784 (0.28%)	106472 (0.16%)	228428 (0.30%)	110562 (0.16%)	255984 (0.31%)	139120 (0.20%)	211792 (0.25%)
With mate mapped to a different chr	167598 (0.21%)	139900 (0.17%)	155144 (0.19%)	139766 (0.21%)	132664 (0.19%)	178160 (0.24%)	168944 (0.25%)	188816 (0.23%)	278236 (0.40%)	153594 (0.18%)
With mate mapped to a different chr ((mapQ≥5))	102905 (0.13%)	87730 (0.11%)	96966 (0.12%)	86808 (0.13%)	82292 (0.12%)	114102 (0.15%)	106929 (0.16%)	116325 (0.14%)	190376 (0.27%)	96325 (0.11%)
Initial_bases_on_target	60456963	60456963	60456963	60456963	60456963	60456963	60456963	60456963	60456963	60456963
Initial_bases_near_target	75840481	75840481	75840481	75840481	75840481	75840481	75840481	75840481	75840481	75840481
Initial_bases_on_or_near_target	136297444	136297444	136297444	136297444	136297444	136297444	136297444	136297444	136297444	136297444
Total_effective_reads	79905860	82526054	82919825	67710961	68340651	75675411	67153887	81588948	69647726	86097911
Total_effective_yield (Mb)	11970.96	12366.48	12424.95	10145.08	10240.61	11339.19	10060.42	12222.78	10432	12901.47
Effective_sequences_on_target (Mb)	7546.78	7952.58	8096.4	6402.72	6639.16	7130.63	6347.48	7664.75	6639.89	8338.81
Effective_sequences_near_target (Mb)	2756.44	2710.35	2627.78	2258.92	2240.49	2575.01	2314.64	2691.35	2375.57	2762.95
Effective_sequences_on_or_near_target (Mb)	10303.22	10662.93	10724.19	8661.64	8879.65	9705.64	8662.12	10356.1	9015.46	11101.75
Fraction_of_effective_bases_on_target	63.04%	64.31%	65.16%	63.11%	64.83%	62.88%	63.09%	62.71%	63.65%	64.63%
Fraction_of_effective_bases_on_or_near_target	86.07%	86.22%	86.31%	85.38%	86.71%	85.59%	86.10%	84.73%	86.42%	86.05%
Average_sequencing_depth_on_target	125	132	134	106	110	118	105	127	110	138
Average_sequencing_depth_near_target	36.35	35.74	34.65	29.79	29.54	33.95	30.52	35.49	31.32	36.43
Mismatch_rate_in_target_region	0.46%	0.61%	0.57%	0.69%	0.48%	0.62%	0.52%	0.71%	0.55%	0.60%
Mismatch_rate_in_all_effective_sequence	0.59%	0.75%	0.71%	0.86%	0.60%	0.79%	0.66%	0.90%	0.70%	0.74%
Base_covered_on_target	60358399	60385259	60389131	60379797	60379235	60385598	60385485	60381500	60390179	60255674
Coverage_of_target_region	99.84%	99.88%	99.89%	99.87%	99.87%	99.88%	99.88%	99.88%	99.89%	99.67%
Base_covered_near_target	74523196	74448064	74574165	74401585	74162435	75026498	74631870	74296504	74620357	74177872
Coverage_of_flanking_region	98.26%	98.16%	98.33%	98.10%	97.79%	98.93%	98.41%	97.96%	98.39%	97.81%
Fraction_of_target_covered_with_at_least_10x	99.06%	99.53%	99.62%	99.43%	99.45%	99.54%	99.38%	99.02%	99.53%	99.15%
Fraction_of_target_covered_with_at_least_50x	85.50%	88.48%	92.34%	83.52%	88.05%	90.10%	80.74%	85.30%	82.62%	87.57%
Fraction_of_target_covered_with_at_least_100x	52.26%	55.08%	61.64%	41.64%	48.79%	52.73%	38.51%	55.50%	42.70%	57.62%
Fraction_of_flanking_region_covered_with_at_least_10x	75.43%	72.30%	71.69%	69.61%	68.26%	75.67%	70.71%	73.20%	71.91%	71.95%
Fraction_of_flanking_region_covered_with_at_least_50x	23.22%	22.87%	23.00%	17.40%	18.74%	22.12%	17.32%	22.87%	18.37%	23.08%
Fraction_of_flanking_region_covered_with_at_least_100x	6.60%	6.60%	6.00%	3.99%	3.70%	4.86%	4.33%	6.54%	4.72%	7.15%
**Sample**	**NR8-N**	**R1-N**	**R3-T**	**NR4-N**	**NR6-T**	**NR1-T**	**R5-N**	**R4-T**	**NR7-N**	
Total	66709478 (100%)	67400714 (100%)	82209618 (100%)	78848736 (100%)	77948406 (100%)	95492716 (100%)	78539690 (100%)	71703714 (100%)	88333692 (100%)	
Duplicate	11885876 (17.82%)	8531265 (12.66%)	15148108 (18.43%)	14120192 (17.91%)	13412309 (17.21%)	14403832 (15.08%)	14214711 (18.10%)	9440179 (13.17%)	11858874 (13.43%)	
Mapped	66656906 (99.92%)	67298408 (99.85%)	82148077 (99.93%)	78790690 (99.93%)	77881525 (99.91%)	95351431 (99.85%)	78414629 (99.84%)	71605615 (99.86%)	88187251 (99.83%)	
Properly mapped	66366104 (99.49%)	66954576 (99.34%)	81700144 (99.38%)	78453204 (99.50%)	77466276 (99.38%)	94885644 (99.36%)	78002074 (99.32%)	71262104 (99.38%)	87752932 (99.34%)	
PE mapped	66616024 (99.86%)	67211022 (99.72%)	82096820 (99.86%)	78746620 (99.87%)	77827624 (99.85%)	95266952 (99.76%)	78347418 (99.76%)	71516788 (99.74%)	88072110 (99.70%)	
SE mapped	81764 (0.12%)	174772 (0.26%)	102514 (0.12%)	88140 (0.11%)	107802 (0.14%)	168958 (0.18%)	134422 (0.17%)	177654 (0.25%)	230282 (0.26%)	
With mate mapped to a different chr	152626 (0.23%)	152568 (0.23%)	200500 (0.24%)	193976 (0.25%)	183536 (0.24%)	177282 (0.19%)	204204 (0.26%)	140078 (0.20%)	220692 (0.25%)	
With mate mapped to a different chr ((mapQ≥5))	92576 (0.14%)	97273 (0.14%)	119800 (0.15%)	118132 (0.15%)	109822 (0.14%)	111455 (0.12%)	126323 (0.16%)	91610 (0.13%)	138171 (0.16%)	
Initial_bases_on_target	60456963	60456963	60456963	60456963	60456963	60456963	60456963	60456963	60456963	
Initial_bases_near_target	75840481	75840481	75840481	75840481	75840481	75840481	75840481	75840481	75840481	
Initial_bases_on_or_near_target	136297444	136297444	136297444	136297444	136297444	136297444	136297444	136297444	136297444	
Total_effective_reads	66767920	67397117	82303730	78932757	78016090	95490365	78542028	71719095	88334208	
Total_effective_yield (Mb)	10003.71	10099.25	12329.5	11825.19	11688.52	14308.81	11767.77	10745.85	13234.95	
Effective_sequences_on_target (Mb)	6483.03	6373.42	7606.15	7546.18	7341.26	9126.55	7499.55	6905.02	8388.34	
Effective_sequences_near_target (Mb)	2267.85	2275.52	2936.72	2655.99	2848.85	3311.61	2709.61	2356.88	2848.92	
Effective_sequences_on_or_near_target (Mb)	8750.88	8648.95	10542.87	10202.17	10190.1	12438.16	10209.16	9261.89	11237.27	
Fraction_of_effective_bases_on_target	64.81%	63.11%	61.69%	63.81%	62.81%	63.78%	63.73%	64.26%	63.38%	
Fraction_of_effective_bases_on_or_near_target	87.48%	85.64%	85.51%	86.27%	87.18%	86.93%	86.76%	86.19%	84.91%	
Average_sequencing_depth_on_target	107	105	126	125	121	151	124	114	139	
Average_sequencing_depth_near_target	29.9	30	38.72	35.02	37.56	43.67	35.73	31.08	37.56	
Mismatch_rate_in_target_region	0.47%	0.59%	0.47%	0.46%	0.50%	0.52%	0.51%	0.61%	0.60%	
Mismatch_rate_in_all_effective_sequence	0.58%	0.75%	0.62%	0.59%	0.63%	0.66%	0.64%	0.76%	0.77%	
Base_covered_on_target	60382028	60253815	60386558	60384621	60390992	60390876	60394741	60239867	60393796	
Coverage_of_target_region	99.88%	99.66%	99.88%	99.88%	99.89%	99.89%	99.90%	99.64%	99.90%	
Base_covered_near_target	74348596	74593614	75231665	74782678	75242850	75138969	74887200	74023672	74860786	
Coverage_of_flanking_region	98.03%	98.36%	99.20%	98.61%	99.21%	99.08%	98.74%	97.60%	98.71%	
Fraction_of_target_covered_with_at_least_10x	99.48%	99.27%	99.60%	99.59%	99.59%	99.61%	99.61%	99.14%	99.65%	
Fraction_of_target_covered_with_at_least_50x	85.85%	87.01%	91.43%	90.55%	90.46%	93.04%	91.26%	85.43%	92.66%	
Fraction_of_target_covered_with_at_least_100x	44.33%	44.10%	56.49%	55.53%	53.60%	66.28%	56.40%	48.71%	63.00%	
Fraction_of_flanking_region_covered_with_at_least_10x	70.20%	71.89%	80.88%	74.61%	80.54%	80.06%	75.89%	69.61%	75.10%	
Fraction_of_flanking_region_covered_with_at_least_50x	18.07%	18.13%	26.35%	22.91%	25.23%	30.23%	24.04%	19.56%	25.25%	
Fraction_of_flanking_region_covered_with_at_least_100x	3.87%	3.54%	6.49%	5.65%	5.96%	9.49%	5.67%	4.61%	6.99%	
**Sample**	**R2-T**	**R2-N**	**R4-N**	**NR2-T**	**R5-T**	**NR3-T**	**NR3-N**			
Total	67883584 (100%)	66842058 (100%)	72641460 (100%)	73005814 (100%)	87833810 (100%)	83586362 (100%)	75497916 (100%)			
Duplicate	9879021 (14.55%)	12008043 (17.96%)	12663288 (17.43%)	10827152 (14.83%)	15338279 (17.46%)	11599176 (13.88%)	11776171 (15.60%)			
Mapped	67787707 (99.86%)	66794473 (99.93%)	72593017 (99.93%)	72940516 (99.91%)	87777226 (99.94%)	83442865 (99.83%)	75384112 (99.85%)			
Properly mapped	67400626 (99.29%)	66495234 (99.48%)	72277378 (99.50%)	72658694 (99.52%)	87332712 (99.43%)	83036784 (99.34%)	75024198 (99.37%)			
PE mapped	67708108 (99.74%)	66757268 (99.87%)	72554938 (99.88%)	72883690 (99.83%)	87732082 (99.88%)	83330776 (99.69%)	75317680 (99.76%)			
SE mapped	159198 (0.23%)	74410 (0.11%)	76158 (0.10%)	113652 (0.16%)	90288 (0.10%)	224178 (0.27%)	132864 (0.18%)			
With mate mapped to a different chr	132844 (0.20%)	166026 (0.25%)	165394 (0.23%)	147554 (0.20%)	205220 (0.23%)	225474 (0.27%)	167026 (0.22%)			
With mate mapped to a different chr ((mapQ≥5))	83795 (0.12%)	101761 (0.15%)	99139 (0.14%)	89661 (0.12%)	124031 (0.14%)	141922 (0.17%)	101796 (0.13%)			
Initial_bases_on_target	60456963	60456963	60456963	60456963	60456963	60456963	60456963	
Initial_bases_near_target	75840481	75840481	75840481	75840481	75840481	75840481	75840481			
Initial_bases_on_or_near_target	136297444	136297444	136297444	136297444	136297444	136297444	136297444			
Total_effective_reads	67893757	66914162	72725835	73062855	87933910	83629093	75503738			
Total_effective_yield (Mb)	10172.91	10024.71	10895.15	10946.52	13173.3	12525.27	11313.17			
Effective_sequences_on_target (Mb)	6585.01	6423.52	6717.68	6740.85	8256.71	8062.33	7338.48			
Effective_sequences_near_target (Mb)	2224.73	2226.58	2584.46	2624.55	2984.25	2580.23	2512.66			
Effective_sequences_on_or_near_target (Mb)	8809.74	8650.1	9302.14	9365.4	11240.96	10642.55	9851.13			
Fraction_of_effective_bases_on_target	64.73%	64.08%	61.66%	61.58%	62.68%	64.37%	64.87%			
Fraction_of_effective_bases_on_or_near_target	86.60%	86.29%	85.38%	85.56%	85.33%	84.97%	87.08%			
Average_sequencing_depth_on_target	109	106	111	111	137	133	121			
Average_sequencing_depth_near_target	29.33	29.36	34.08	34.61	39.35	34.02	33.13			
Mismatch_rate_in_target_region	0.58%	0.46%	0.46%	0.53%	0.45%	0.67%	0.51%			
Mismatch_rate_in_all_effective_sequence	0.72%	0.58%	0.61%	0.68%	0.59%	0.84%	0.63%			
Base_covered_on_target	60383718	60383088	60251686	60383299	60389822	60383167	60385263			
Coverage_of_target_region	99.88%	99.88%	99.66%	99.88%	99.89%	99.88%	99.88%			
Base_covered_near_target	74308999	74342915	74882878	74906759	74915786	74129692	74535715			
Coverage_of_flanking_region	97.98%	98.03%	98.74%	98.77%	98.78%	97.74%	98.28%			
Fraction_of_target_covered_with_at_least_10x	99.38%	99.48%	99.30%	99.32%	99.56%	99.26%	99.57%			
Fraction_of_target_covered_with_at_least_50x	81.85%	85.42%	88.31%	85.20%	90.30%	87.08%	90.21%			
Fraction_of_target_covered_with_at_least_100x	41.81%	43.32%	47.62%	47.63%	57.38%	57.76%	54.01%			
Fraction_of_flanking_region_covered_with_at_least_10x	67.73%	69.54%	77.73%	77.99%	76.76%	69.81%	71.84%			
Fraction_of_flanking_region_covered_with_at_least_50x	16.77%	17.41%	21.79%	22.11%	25.76%	21.38%	21.54%			
Fraction_of_flanking_region_covered_with_at_least_100x	4.29%	3.74%	4.59%	5.06%	7.50%	6.40%	5.12%			

**Supplement Table 4:** All somatic single nucleotide variant identified in discovery cohort

Sample	NR7	NR5	NR4	NR8	R1	R3	NR6	NR1	R4	R2	NR2	R5	NR3
CDS	112	202	276	19	189	10	48	61	152	335	155	320	87
synonymous_SNP	30	53	59	5	49	6	24	17	38	92	38	91	28
missense_SNP	69	136	194	12	126	4	24	38	97	211	100	208	48
stopgain	12	6	15	1	4	0	0	5	13	18	11	11	7
stoploss	0	1	1	0	1	0	0	0	0	1	0	1	0
unknown	1	6	7	1	9	0	0	1	4	13	6	9	4
intronic	219	389	359	50	348	53	267	144	330	497	245	628	100
UTR3	13	39	34	2	22	3	14	3	18	35	14	33	11
UTR5	16	22	20	3	18	0	8	2	13	26	17	37	12
splicing	6	9	10	1	5	0	1	2	5	10	6	9	4
ncRNA_exonic	13	19	10	6	16	1	12	12	17	22	4	21	9
ncRNA_intronic	20	33	45	27	37	21	48	24	32	47	25	46	19
ncRNA_UTR3	0	0	0	0	0	0	0	0	0	0	0	0	0
ncRNA_UTR5	0	0	0	0	0	0	0	0	0	0	0	0	0
ncRNA_splicing	0	1	0	0	0	0	0	0	1	1	0	0	0
upstream	10	11	14	3	9	2	15	2	14	19	8	37	2
downstream	3	3	0	1	8	1	6	3	3	3	2	10	5
intergenic	126	122	126	82	133	87	110	101	124	136	100	146	72
Total	539	852	897	195	787	178	529	354	709	1131	577	1288	322

**Supplement Table 5:** The somatic indels identified in discovery cohort

Sample	NR7	NR5	NR4	NR8	R1	R3	NR6	NR1	R4	R2	NR2	R5	NR3
CDS	5	8	10	2	18	1	3	6	4	5	11	21	1
frameshift_deletion	1	4	6	1	6	1	1	5	3	3	5	12	0
frameshift_insertion	2	2	2	1	2	0	1	0	0	2	4	3	0
nonframeshift_deletion	2	2	1	0	9	0	0	0	1	0	0	4	1
nonframeshift_insertion	0	0	0	0	0	0	0	1	0	0	2	1	0
stopgain	0	0	0	0	0	0	1	0	0	0	0	1	0
stoploss	0	0	0	0	0	0	0	0	0	0	0	0	0
unknown	0	0	1	0	1	0	0	0	0	0	0	0	0
intronic	13	14	14	2	17	0	25	7	1	7	4	25	0
UTR3	0	0	2	0	0	0	0	1	1	0	1	2	0
UTR5	0	0	0	0	2	0	2	0	0	1	1	3	0
splicing	0	1	0	0	0	0	0	0	0	0	0	0	0
ncRNA_exonic	0	1	1	0	0	0	0	0	1	0	0	1	0
ncRNA_intronic	1	0	0	0	0	0	0	0	0	1	1	2	0
ncRNA_UTR3	0	0	0	0	0	0	0	0	0	0	0	0	0
ncRNA_UTR5	0	0	0	0	0	0	0	0	0	0	0	0	0
ncRNA_splicing	0	0	0	0	0	0	0	0	0	0	0	0	0
upstream	0	0	1	0	1	0	1	0	0	0	0	2	0
downstream	0	1	0	0	0	0	0	0	0	0	0	0	0
intergenic	2	2	3	0	2	0	3	1	0	0	1	5	0
Total	21	27	31	4	40	1	34	15	7	14	19	61	1

**Supplement Table 6:** All Somatic mutation identified in discovery cohort

Gene	Total	NR2	R4	NR5	R2
*TP53*	7	1 (Missense_Mutation#17:7577538 #rs11540652#C>T)	1 (Frame_Shift_Del#17:757 4029#.#CG>C)	1 (Missense_Mutation# 17:7577085#rs 112431538#C>T)	1 (Missense_Mutation#17:7578406# rs28934578#C>T)	
*KMT2D*	4	0	0	2 (Missense_Mutation#12: 49420600#.#A>G; In_ Frame_Del#12:494265 15#.#CTGT>C)	2 (Nonsense_Mutation# 12:49425545#.#G>A; Nonsense_Mutation# 12:49438595#.#G>T)
*ADAMTS12*	3	0	0	0	0
*PKHD1L1*	3	1 (Missense_Mutation#8: 110489456#.#T>C)	0	0	0
*RB1*	3	1 (Nonsense_Mutation#13:48 947596#.#C>T)	1 (Splice_Site#13:49047 529#.#G>C)	0	0
*TTN*	3	2 (Missense_Mutation#2: 179413188#.#T>A; Missense_Mutation#2: 179616442#.#G>A)	0	0	0
*MED16*	3	0	0	1 (Missense_Mutation# 19:881678#.#A>G)	0
*HYDIN*	3	0	0	0	1 (Missense_Mutation# 16:70998736#.#C>T)
*GTF2IRD1*	3	1 (Missense_Mutation#7: 74005310#.#G>A)	0	0	1 (Missense_Mutation# 7:73954226#.#G>A)
*CROCC*	3	1 (Missense_Mutation#1: 17274844#.#G>A)	0	0	0
*AHNAK2*	3	0	0	0	0
*ASB10*	2	0	0	1 (Missense_Mutation#7:15 0878324#.#G>C)	0
*TMC5*	2	0	0	0	2 (Missense_Mutation# 16:19468122#.#C>G;
*KCTD1*	2	0	0	0	Missense_Mutation# 16:19468165#.#C>T)
*USH2A*	2	0	0	0	0
*LAMA1*	2	0	0	0	0
*IKBIP*	2	0	0	1 (Missense_Mutation# 12:99007505#.#C>T)	1 (Missense_Mutation#12:99007458#.#C>T)
*ZFHX4*	2	0	0	0	1 (Nonsense_Mutation#8:77767954#.#C>T)
*ADAMTS16*	2	0	0	0	0
*TMEM132D*	2	0	0	0	0
*CAPN15*	2	0	0	0	0
*GABRA2*	2	0	1 (Missense_Mutation#4:4631 2262#.#G>C)	0	0
*PLXNB2*	2	0	0	0	1 (Missense_Mutation#22:50728769#.#A>G)
*CCDC168*	2	0	0	0	0
*TMTC2*	2	0	0	1 (Missense_Mutation#12:83 290170#.#A>G)	0
*MAP3K1*	2	0	0	1 (Missense_Mutation#5: 56160646#.#G>A)	1 (Missense_Mutation#5:56155587#.#C>G)
*PZP*	2	0	0	1 (Missense_Mutation#12: 9307408#.#T>C)	0
*TSPEAR*	2	0	0	0	0
*POLD2*	2	1 (Missense_Mutation# 7:44154964#.#G>T)	0	0	0
*SLC12A6*	2	0	0	0	0
*RGS3*	2	0	0	0	0
*SETX*	2	0	0	0	1 (Missense_Mutation#9:135203185#.#G>A)
*FAM135B*	2	0	0	0	0
*PDZD2*	2	0	0	0	0
*UTP6*	2	0	0	0	0
*KIF16B*	2	0	0	1 (Missense_Mutation#20:16 359862#.#C>G)	0
*CRISPLD1*	2	0	0	0	1 (Missense_Mutation#8:75925219#.#T>C)
*KRTAP2-3*	2	0	0	0	0
*EPN3*	2	1 (Frame_Shift_Del#17:48619468#. #GCCGGGCCGCGGCCC>G)	0	0	0
*SETD8*	2	0	0	0	0
*TTBK1*	2	1 (Missense_Mutation# 6:43251852#.#C>T)	0	0	1 (Missense_Mutation#6:43220570#.#C>A)
*MUC16*	2	0	0	0	1 (Missense_Mutation#19:9069457#.#C>T)
*EP300*	2	1 (Missense_Mutation#22:41565575#.#A>G)	1 (Frame_Shift_Del#22:415 46157#.#TC>T)	0	0
*OR2T2*	2	1 (Frame_Shift_Del#1:248616704#rs 199823862#CTGCTGCG>C)	0	0	0
*FAM181B*	2	0	0	1 (Missense_Mutation#11: 82444658#.#G>C)	0
*SIPA1L2*	2	0	0	0	1 (Missense_Mutation#1:232579352#.#C>G)
*ATAD5*	2	0	0	0	0
*ARID1A*	2	1 (Frame_Shift_Ins#1:27023743#.#C>CG)	1 (Nonsense_Mutation#1:27 088697#.#C>G)	0	0
*NOTCH1*	2	0	0	0	0
*ASAP1*	2	0	0	1 (Missense_Mutation#8: 131104250#.#A>T)	1 (Missense_Mutation#8:131414177#.#C>T)
*PDE4DIP*	2	0	0	1 (Missense_Mutation#1:1448 86200#.#C>G)	0
*STARD9*	2	1 (Missense_Mutation#15:42981472#.#C>T)	0	0	0
*ARHGAP35*	2	0	0	0	1 (Nonsense_Mutation#19:47423901#.#C>T)
*GOLGA8K*	2	0	0	0	0
*COL6A3*	2	0	0	1 (Missense_Mutation#2:238 275617#.#C>A)	0
*CCND3*	2	0	0	0	1 (Missense_Mutation#6:41905106#.#C>A)
*COL6A6*	2	0	1 (Missense_Mutation#3: 130311547#.#G>A)	1 (Missense_Mutation#3:1303 00867#.#G>A)	0
*TP53*		0	0	1 (Missense_Mutation#17: 757708 5#rs112431538#C>T)	0
*KMT2D*		1 (Missense_Mutation# 12:49439936#.#C>G)	0	0	0
*ADAMTS12*		0	1 (Missense_Mutation#5: 33881369#.#T>C)	0	1 (Missense_Mutation#5:33577132# rs13362345#C>T)
*PKHD1L1*		1 (Missense_Mutation# 8:110460413#.#G>T)	1 (Missense_Mutation#8: 110457541#.#G>A)	0	0
*RB1*		0	0	1 (Frame_Shift_Del#13: 48878126#.#GC>G)	0
*TTN*		0	1 (Missense_Mutation#2: 179506013#.#G>C)	0	0
*MED16*		0	0	0	0
*HYDIN*		0	0	1 (Missense_Mutation#16: 71019216#.#G>T)	0
*GTF2IRD1*		0	0	0	0
*CROCC*		0	1 (Missense_Mutation#1: 17263208#.#G>T)	0	0
*AHNAK2*		0	1 (Missense_Mutation#14: 105420326#.#C>A)	0	1 (Nonsense_Mutation#14:105423815#. #G>A)
*ASB10*		0	1 (Missense_Mutation#7: 150883650#.#C>T)	0	0
*TMC5*		0	0	1 (Missense_Mutation#16: 19475128#.#G>C)	0
*KCTD1*		0	1 (Missense_Mutation#18: 24127513#.#C>T)	0	0
*USH2A*		0	1 (Missense_Mutation#1: 216166443#rs375278546#C>T)	0	0
*LAMA1*		1 (Missense_Mutation# 18:6977821#rs146111631#G>A)	0	0	0
*IKBIP*		0	0	0	0
*ZFHX4*		0	0	1 (Nonsense_Mutation#8: 77617248#.#G>T)	0
*ADAMTS16*		1 (Frame_Shift_Ins# 5:5262847#.#C>CAG)	1 (Missense_Mutation#5:51463 20#rs375714169#C>T)	0	0
*TMEM132D*		0	1 (Missense_Mutation#12: 130184368#.#G>T)	1 (Missense_Mutation#12: 129559454#.#T>C)	0
*CAPN15*		0	1 (Missense_Mutation#16: 598178#.#G>A)	2 (Missense_Mutation#16: 596969#.#A>G; Missense_Mutation#16:596970#.#G>T)	0
*GABRA2*		0	0	0	1 (Missense_Mutation#4:46305489#.#T>C)
*PLXNB2*		0	0	1 (Frame_Shift_Del#22: 50715101#.#AC>A)	0
*CCDC168*		0	1 (Missense_Mutation#13: 103384106#.#G>C)	1 (Missense_Mutation#13: 103385255#.#G>A)	0
*TMTC2*		0	0	0	0
*MAP3K1*		0	0	0	0
*PZP*		0	1 (Missense_Mutation#12:93552 19#rs142943281#G>A)	0	0
*TSPEAR*		0	1 (Missense_Mutation #21:45945689#.#C>T)	0	0
*POLD2*		0	0	0	0
*SLC12A6*		0	1 (Missense_Mutation#15: 34628716#.#G>C)	1 (Missense_Mutation#15: 34551139#.#T>C)	0
*RGS3*		0	1 (Missense_Mutation#9: 116222616#.#G>A)	0	0
*SETX*		1 (Missense_Mutation#9:135156856#.#T>C)	0	0	0
*FAM135B*		0	1 (Missense_Mutation#8:1391640 65#rs570924723#C>T)	1 (Missense_Mutation#8: 139164311#.#T>A)	0
*PDZD2*		0	1 (Missense_Mutation#5:320 74252#rs61745924#G>A)	1 (Missense_Mutation#5: 32090663#.#G>A)	0
*UTP6*		0	1 (Missense_Mutation# 17:30190461#.#A>G)	0	0
*KIF16B*		0	1 (Missense_Mutation# 20:16387066#.#G>A)	0	0
*CRISPLD1*		0	0	0	0
*KRTAP2-3*		0	1(Missense_Mutation#17:3921 6128#rs35027423#G>A)	0	0
*EPN3*		0	1 (Splice_Site#17:48610349#.#A>G)	0	0
*SETD8*		0	0	0	1 (Missense_Mutation#12: 123889486#rs61955123#G>C)
*TTBK1*		0	0	0	0
*MUC16*		0	0	1 (Missense_Mutation#19 :9088555#.#G>C)	0
*EP300*		0	0	0	0
*OR2T2*		0	0	0	0
*FAM181B*		0	0	0	0
*SIPA1L2*		1 (Missense_Mutation# 1:232564258#.#C>G)	0	0	0
*ATAD5*		0	1 (Missense_Mutation#17: 29171019#.#T>G)	0	0
*ARID1A*		0	0	0	0
*NOTCH1*		1 (Missense_Mutation# 9:139390743#.#G>A)	1 (Missense_Mutation#9: 139390600#.#C>A)	0	0
*ASAP1*		0	0	0	0
*PDE4DIP*		0	0	0	0
*STARD9*		0	1 (Missense_Mutation#15: 42985917#.#G>C)	0	0
*ARHGAP35*		0	0	0	0
*GOLGA8K*		0	0	0	0
*COL6A3*		0	0	0	0
*CCND3*		0	0	0	1 (Nonsense_Mutation#6:41903779#.#G>A)
*COL6A6*		0	0	0	0

***Gene***	**NR8**	**R1**	**R3**	**NR6**	**NR1**

*TP53*	1 (Missense_Mutation# 17:7578190#rs121912666#T>C)	0	0	1 (Missense_Mutation# 17:7578190#rs121912666#T>C)	0
*KMT2D*	0	0	0	0	1(Missense_Mutation#12:49 441813#.#C>T)
*ADAMTS12*	0	0	0	0	0
*PKHD1L1*	0	0	0	1(Missense_Mutation#5:338812 52#rs117518215#G>A)	0
*RB1*	0	0	0	0	0
*TTN*	0	0	0	0	0
*MED16*	2 (Frame_Shift_Del# 19:875274#.#TCAGCC>T; Frame_Shift_Ins# 19:875295#.#T>TTAAAAAA)	1 (Frame_Shift_Ins# 19:875295#.#T>TTAAAAAA)	0	0	0
*HYDIN*	0	1 (Missense_Mutation# 16:71186679#.#A>C)	0	0	0
*GTF2IRD1*	0	1 (In_FrameDel#7:73961544#.# CCAACTGCTTCGGGAT>C)	0	0	0
*CROCC*	0	1 (Missense_Mutation# 1:17256695#.#G>T)	0	0	0
*AHNAK2*	0	1 (Missense_Mutation# 14:105409138#.#G>C)	0	0	0
*ASB10*	0	0	0	0	0
*TMC5*	0	0	0	0	0
*KCTD1*	0	0	0	0	1(Missense_Mutation#18:2 4127091#.#C>A)
*USH2A*	0	0	0	0	1(Missense_Mutation#1:21 6465538#.#C>A)
*LAMA1*	0	1 (Missense_Mutation# 18:7080061#.#T>C)	0	0	0
*IKBIP*	0	0	0	0	0
*ZFHX4*	0	0	0	0	0
*ADAMTS16*	0	0	0	0	0
*TMEM132D*	0	0	0	0	0
*CAPN15*	0	0	0	0	0
*GABRA2*	0	0	0	0	0
*PLXNB2*	0	0	0	0	0
*CCDC168*	0	0	0	0	0
*TMTC2*	0	1 (In_Frame_Del#12:83290305#. #ATTTTTTATGCTACAG CTACACTAATTG>A)	0	0	0
*MAP3K1*	0	0	0	0	0
*PZP*	0	0	0	0	0
*TSPEAR*	0	0	0	0	1(Missense_Mutation#21:4 5945556#.#G>A)
*POLD2*	0	1 (Missense_Mutation#7: 44155843#.#C>A)	0	0	0
*SLC12A6*	0	0	0	0	1(Missense_Mutation#9:1162 59672#.#C>T)
*RGS3*	0	0	0	0	0
*SETX*	0	0	0	0	0
*FAM135B*	0	0	0	0	0
*PDZD2*	0	0	0	0	0
*UTP6*	0	0	0	1(Missense_Mutation#17:30222 002#rs3760454#T>C)	0
*KIF16B*	0	0	0	0	0
*CRISPLD1*	0	0	0	0	1(Missense_Mutation#8: 75929320#.#A>T)
*KRTAP2-3*	0	0	0	1(Missense_Mutation#17:39216 085#rs113397060#C>T)	0
*EPN3*	0	0	0	0	0
*SETD8*	0	0	2 (Missense_Mutation#12: 123879666#rs61955119# A>G;Missense_Mutation#12 :123879668#rs61955120#G>C)	0	0
*TTBK1*	0	0	0	0	0
*MUC16*	0	0	0	0	0
*EP300*	0	0	0	0	0
*OR2T2*	0	0	1 (Frame_Shift_Del#1:248616704#rs 199823862#CTGCTGCG>C)	0	0
*FAM181B*	0	1 (Missense_Mutation#11: 82443571#.#G>C)	0	0	1 (Missense_Mutation# 11:82443571#.#G>C)
*SIPA1L2*	0	0	0	3(Missense_Mutation#17:29161 202#rs9910051#A>T;Missense _Mutation# 17:29167653#rs3764421 #A>C;Missense_Mutation#17:292143 87#rs11657270#T>C)	0
*ATAD5*	0	0	0	0	0
*ARID1A*	0	0	0	0	0
*NOTCH1*	0	0	0	0	0
*ASAP1*	0	0	0	0	0
*PDE4DIP*	0	1 (Missense_Mutation#1:14485 6817#rs3844239#T>C)	0	0	1 (Missense_Mutation# 1:144856817#rs3844239#T>C)
*STARD9*	0	0	0	0	0
*ARHGAP35*	1 (Missense_Mutation# 19:47425573#.#G>C)	0	0	0	0
*GOLGA8K*	0	1 (Missense_Mutation#15:3268 8657#rs372059899#T>G)	0	1 (Missense_Mutation#19: 47425573#.#G>C)	1 (Missense_Mutation# 15:32688657#rs372059899#T>G)
*COL6A3*	0	1 (Missense_Mutation#2: 238287506#.#T>A)	0	0	1 (Missense_Mutation# 2:238287506#.#T>A)
*CCND3*	0	0	0	0	0
*COL6A6*	0	0	0	0	0

**Supplementary Table 7:** Significantly mutated genes of 13 bladder cancer patients

#Gene	Indels	SNVs	Tot Muts	Sample No.	Sample Percent (%)	P-value	FDR
*TP53*	2	5	7	7	53.85	1.72E-14	3.29E-10
*MED16*	3	1	4	3	23.08	2.33E-08	2.23E-04
*DRC7*	0	5	5	1	7.69	3.92E-08	2.50E-04
*CEND1*	1	2	3	1	7.69	8.50E-07	0.004
*ATAD5*	0	4	4	2	15.38	3.49E-06	0.011
*SETD8*	0	3	3	2	15.38	3.52E-06	0.011
*PIK3CA*	0	4	4	3	23.08	4.89E-06	0.013

**Supplementary Figure 2:**
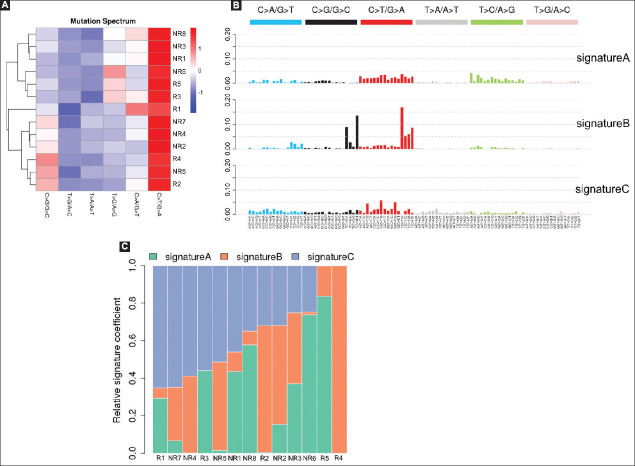
Spectrum of somatic point mutations identified with the 13 muscle-invasive bladder cancer samples. (A) A mutation spectrum heatmap of 13 muscle-invasive bladder cancer samples. (B) Three mutation signatures identified in the 13 muscle-invasive bladder cancer samples. (C) The contributions of mutation signature A-C in each of the 13 muscle-invasive bladder cancer samples.

The C->T/G->A mutation dominated the mutation spectrum in 13 MIBC samples (Supplementary Figure 2A), and three major mutational signatures (A, B, and C) were identified in 13 MIBC samples (Supplementary Figure 2B and C and Supplementary Table 8). Refer to Signatures of mutational processes in Human Cancer (https://cancer.sanger.ac.uk/cosmic/signatures). The three signatures, A, B, and C, were similar to Single Base Substitution (SBS) Signature 5, SBS Signature 2, and SBS Signature 6, respectively (Supplementary Table 8). Specifically, the contribution of each signature was calculated for each group, and none of the signatures was significantly enriched in nonresponders or responders (Supplementary Table 9)

**Supplementary Table 8:** Mutational signatures of 13 bladder cancer patients

Signature	Near reference signature	Cosine similarity	Correlation coefficient	Filter with cosine similarity >0.9	Cancer types	Proposed aetiology	Additional mutational features	Comments
Signature.A	Signature.5	0.896520855	0.75876711	not pass	Signature 5 has been found in all cancer types and most cancer samples	Signature 5 has been found in all cancer types and most cancer samples	Signature 5 exhibits transcriptional strand bias for T>C substitutions at ApTpN context	N/A
Signature.B	Signature.2	0.835048422	0.83538391	not pass	Signature 2 has been found in 22 cancer types, but most commonly in cervical and bladder cancers. In most of these 22 cancer types, Signature 2 is present in at least 10% of samples	Signature 2 has been attributed to activity of the AID/APOBEC family of cytidine deaminases. On the basis of similarities in the sequence context of cytosine mutations caused by APOBEC enzymes in experimental systems, a role for APOBEC1, APOBEC3A and/or APOBEC3B in human cancer appears more likely than for other members of the family	Transcriptional strand bias of mutations has been observed in exons, but is not present or is weaker in introns	Signature 2 is usually found in the same samples as Signature 13. It has been proposed that activation of AID/APOBEC cytidine deaminases is due to viral infection, retrotransposon jumping or to tissue inflammation. Currently, there is limited evidence to support these hypotheses. A germline deletion polymorphism involving APOBEC3A and APOBEC3B is associated with the presence of large numbers of Signature 2 and 13 mutations and with predisposition to breast cancer. Mutations of similar patterns to Signatures 2 and 13 are commonly found in the phenomenon of local hypermutation present in some cancers, known as kataegis, potentially implicating AID/APOBEC enzymes in this process as well
Signature.C	Signature.6	0.775364877	0.76032566	not pass	Signature 6 has been found in 17 cancer types and is most common in colorectal and uterine cancers. In most other cancer types, Signature 6 is found in less than 3% of examined samples	Signature 6 is associated with defective DNA mismatch repair and is found in microsatellite unstable tumors	Signature 6 is associated with high numbers of small (shorter than 3bp) insertions and deletions at mono/polynucleotide repeats	Signature 6 is one of four mutational signatures associated with defective DNA mismatch repair and is often found in the same samples as Signatures 15, 20, and 26

**Supplementary Table 9:** Mutational signatures analysis in the responder and nonresponder group

	NR1	NR2	NR3	NR4	NR5	NR6	NR7	NR8	R1	R2	R3	R4	R5	p value
Signature A	0.436046512	0.152492669	0.372093023	0	0.020348837	0.738372093	0.067055394	0.574344023	0.289473684	0.011661808	0.438596491	0	0.839181287	0.90715408
Signature B	0.101744186	0.530791789	0.377906977	0.406432749	0.470930233	0.01744186	0.282798834	0.075801749	0.058479532	0.670553936	0	1	0.160818713	0.597282207
Signature C	0.462209302	0.316715543	0.25	0.593567251	0.50872093	0.244186047	0.650145773	0.349854227	0.652046784	0.317784257	0.561403509	0	0	0.379617223

### 3.2. The somatic mutations exclusively occurring in NAC responders or nonresponders in MIBC patients

**Figure 2:**
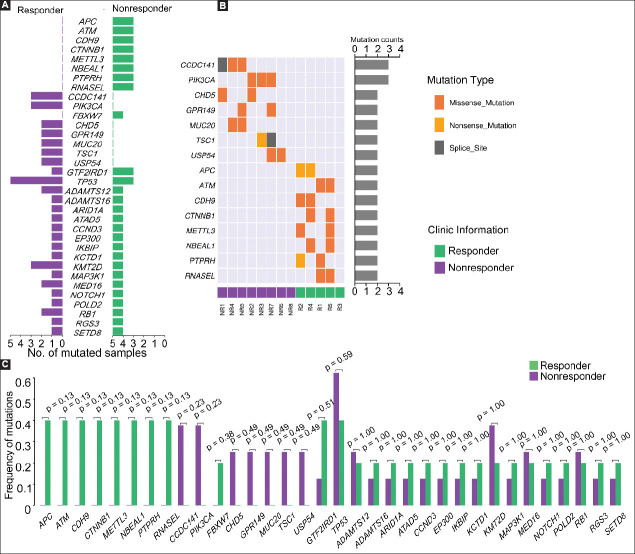
Somatic mutations exclusively occurring in NAC responders or nonresponders in MIBC patients. (A) The somatic mutation rates of key genes in the discovery cohort (*n*=13). (B) The somatic mutations that occur exclusively in the responders (*n*=5) and the nonresponders (*n*=8). Each column represents a tumor, and each row represents a gene. Genes were listed on the left and the center panel is divided into responders (R, green) and nonresponders (NR, purple). The mutation counts were summarized on the right. (C) *APC*, *ATM*, *CDH9*, *CTNNB1*, *METTL3*, *NBEAL1*, *PTPRH*, and *FBXW7* somatic mutations exclusively occur in NAC responders, and *CCDC141*, *PIK3CA*, *CHD5*, *GPR149*, *MUC20*, *TSC1*, and *USP54* somatic mutations exclusively occur in NAC nonresponders. n, patient number.

To determine the differences in mutated genes between NAC responders and nonresponders, genes with different mutation frequencies were studied. In the discovery cohort, the mutations of nine genes (*APC*, *ATM*, *CDH9*, *CTNNB1*, *METTL3*, *NBEAL1*, *PTPRH*, *RNASEL*, and *FBXW7*) were exclusively present in NAC responders (Figure 2A and Supplementary Table 10). However, the NAC nonresponders were exclusively associated with somatic mutations in seven genes (*CCDC141*, *PIK3CA*, *CHD5*, *GPR149*, *MUC20*, *TSC1*, and *USP54*) (Figure 2A and Supplementary Table 11). In addition, somatic mutations of *ADAMTS12*, *ADAMTS16*, *ARID1A*, *ATAD5*, *CCND3*, *EP300*, *IKBIP*, *KCTD1*, *KMY2D*, *MAP3K1*, *MED16*, *NOTCH1*, *POLD2*, *RB1*, *RGS3*, and *SETD8* were identified in both groups. The exclusively mutated genes and type of mutations among NAC responders and nonresponders were depicted in heat map (Figure 2B). Missense mutations were majorly detected in MIBC patients. Nonetheless, based on a mutational analysis, nonsense mutation of *APC* was detected in NAC responders (Figure 2B). However, there were no significant differences in the exclusively mutated genes between NAC responders and nonresponders due to the lack of viable MIBC samples in the discovery cohort (Figure 2C).

**Supplement Table 10:** Specific somatic mutations identified in the responder group in the discovery cohort

Gene	Total	R4	R3	R2	R5	R1
*RNASEL*	2	0	0	0	1 (Missense_Mutation#1:182555491#.#C>T)	1 (Missense_Mutation#1:182555809#.#G>C)
*NBEAL1*	2	1 (Missense_Mutation#2: 204009786#.#A>G)	0	0	1 (Missense_Mutation#2:203972514#.#A>C)	0
*CTNNB1*	2	1 (Missense_Mutation#3: 41278137#.#G>C)	0	0	1 (Missense_Mutation#3:41266450#.#G>A)	0
*CDH9*	2	1 (Missense_Mutation#5: 26885861#.#C>T)	0	1 (Missense_Mutation#5:26988395#.#A>C)	0	0
*APC*	2	1 (Nonsense_Mutation#5: 112154991#.#G>A)	0	1 (Nonsense_Mutation#5:112174437#.#G>A)	0	0
*ATM*	2	0	0	0	2 (Nonsense_Mutation#11:108165741#.#G>T; Missense_Mutation#11:108206609#.#A>G)	1 (Missense_Mutation#11:108155034#.#A>C)
*METTL3*	2	0	0	1 (Missense_Mutation#14:21967704#.#G>C)	1 (Missense_Mutation#14:21971651#.#C>T)	0
*PTPRH*	2	0	0	1(Nonsense_Mutation#19:55693222#.#G>T)	0	1 (Missense_Mutation#19:55693503#.#T>A)
*FBXW7*	1	0	0	0	3 (Missense_Mutation#4:153271228#.#C>G; Frame_Shift_Del#4:153247170#.#GACTCTATTAGTATGCCC>G; In_Frame_Del#4:153253792#.#AAAATTCTCCAGT>A)	0

**Supplement Table 11:** Specific somatic mutations identified in the nonresponder group in the discovery cohort

Gene	Total	NR4	NR7	NR2	NR8	NR5	NR3	NR1	NR6
*CCDC141*	3	1 (Missense_Mutation#2: 179839888#.#G>C)	0	0	0	1 (Missense_Mutation#2: 179698970#.#C>G)	0	1 (Splice_Site#2: 179733841#.#T>C)	0
*PIK3CA*	3	0	2 (Missense_Mutation#3: 178928076#.#T>A; Missense_Mutation#3: 178928079#.#G>A)	1 (Missense_Mutation#3: 178936091#rs 104886003#G>A)	0	0	1 (Missense_Mutation#3: 178951968#.#C>G)	0	0
*TSC1*	2	0	1 (Splice_Site#9: 135802693#.#T>A)	0	0	0	1 (Nonsense_Mutation#9: 135781467#rs 118203537#G>A)	0	0
*USP54*	2	0	1 (Missense_Mutation#10: 75283383#.#G>A)	0	0	0	0	0	1 (Missense_Mutation#10: 75276139#.#G>T)
*MUC20*	2	1 (Missense_Mutation#3: 195452843#rs 370231852#G>A)	0	0	0	1 (Missense_Mutation#3: 195452592#rs 568398932#C>T)	0	0	0
*CHD5*	2	0	0	1 (Missense_Mutation#1: 6206426#.#C>T)	0	0	0	1 (Missense_Mutation#1: 6185655#.#G>A)	0
*GPR149*	2	0	1 (Missense_Mutation#3: 154146882#.#A>C)	0	0	1 (Missense_Mutation#3: 154055736#.#G>C)	0	0	0

Mutations in some of the key genes that have been previously reported as predictive biomarkers of chemotherapy response in BC, such as DNA damage repair (DDR) genes *ERCC2*, *ATM*, *RB1*, and *FANCC*), *FGFR3, ERBB2*, and *BRCA2*, were also examined. In this study, *ATM* mutations were found in 2/21 responders and 0/12 nonresponders (Table 1, *P*=0.27), *RB1* mutations in 1/5 responders and 2/8 nonresponders (Table 1, *P*=0.83), and *FANCC* mutations in 0/5 responders and 1/8 nonresponders (Table 1, *P*=0.41). However, the mutation of *BRCA2* was not detected in this study. Furthermore, *FGFR3* mutations were found in 0/5 responders and 1/8 nonresponders (Table 1, *P*=0.41), *ERBB2* mutations in 0/5 responders and 1/8 nonresponders (Table 1, *P*=0.41), and *ERCC2* mutations in 1/5 responders and 1/8 nonresponders (Table 1, *P*=0.72). The differences in races, treatment methods and sample sizes might account for this inconsistency. In view of this, the somatic mutations exclusively found in the NAC responders and nonresponders were further examined in the validation cohort.

### 3.3. CDH9, METTL3, PTPRH, and CCDC141 somatic mutations were significantly enriched in the validation cohort

To further validate our findings, we compared the somatic mutation frequencies of the 16 exclusively mutated genes in the validation cohort (*n*=20). We detected the presence of somatic mutations in *CDH9 (*7/16), *METTL3 (*6/16), *PTPRH* (5/16), and *CCDC141 (*2/4) in the validation cohort (Table 1). Combined with discovery cohort (*n*=33), there were 12 nonresponders and 21 responders (Table 1). Interestingly, *CDH9 (*9/21, *P*=0.008), *METTL3 (*8/21, *P*=0.014), *PTPRH* (7/21, *P*=0.024), and *CCDC141 (*5/12, *P*=0.013) exhibited significant differences in mutation frequencies between NAC nonresponders and responders (Table 1).

**Figure 3:**
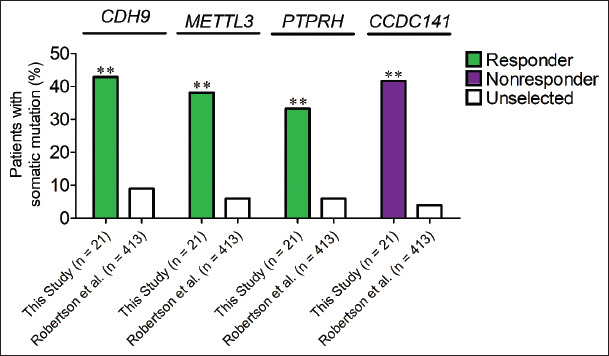
*CDH9*, *METTL3*, *PTPRH*, and *CCDC141* somatic mutations were significantly enriched in the validation cohort. *CDH9*, *METTL3*, and *PTPRH* somatic mutations were significantly enriched in the NAC responders as compared to the unselected urothelial carcinoma cohort (Robertson *et al.*, 2017). *CCDC141* somatic mutations were significantly enriched in NAC nonresponders as compared to the unselected urothelial carcinoma cohort (Robertson *et al.*, 2017).

The somatic mutation frequencies of *CDH9*, *METTL3*, and *PTPRH* in the responder group and *CCDC141* in the nonresponder group were also compared with those in the unselected BC cohorts [17]. Remarkably, the somatic mutations of *CDH9*, *METTL3*, and *PTPRH* were significantly enriched in NAC responders as compared to the unselected BC patients (Figure 3, *P*<0.01). Apart from that, NAC nonresponders had significantly higher *CCDC141* somatic mutation frequencies as compared to the unselected BC patients (Figure 3, *P*<0.01). According to the data from the study of Van Allen *et al.*, *METTL3* was found to be exclusively mutated in the responder group (2/25) and *CCDC141* was exclusively mutated in the nonresponder group (1/25) (Table 2). However, *PTPRH* was mutated in the both responder group (1/25) and the nonresponder group (1/25) and no somatic mutations were detected in *CDH9* gene (Table 2). Unfortunately, there were no significant differences between these two groups due to the small number of samples. Taken together, these results suggested that *CDH9*, *METTL3*, and *PTPRH* somatic mutations were probably associated with NAC response, while *CCDC141* mutation was probably associated with resistance to NAC.

**Table 2:** Mutation frequencies of CDH9, METTL3, PTPRH, and CCDC141 in Van Allen dataset and this study

	Study	Total (33)	Nonresponders	Responders	*P* value
*CDH9*	This study	9	0/12 0	9/21	0.008
*METTL3*		8	0/12 0	8/21	0.014
*PTPRH*		7	0/12 0	7/21	0.024
*CCDC141*		5	5/12	0/21 0	0.013
*CDH9*	Van Allen *et al*. (13)	0	0/25	0/25	1.000
*METTL3*		2	0/25	2/25	0.149
*PTPRH*		2	1/25	1/25	1.000
*CCDC141*		1	1/25	0/25	0.312

### 3.4. METTL3 mutation predicts better prognosis of BC patients

**Figure 4:**
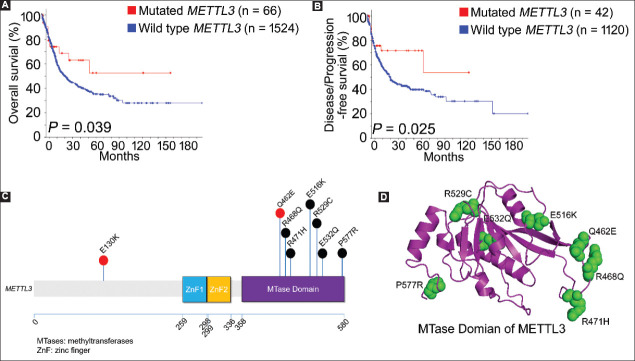
*METTL3* mutation predicts NAC response in MIBC patients. (A) A stick plot of *METTL3* showing the locations of mutations in the MIBC samples. Black, reported somatic mutations. Red, newly identified somatic mutations. (B) Structure of the methyltransferase domain of METTL3 (PDB code, 5IL0) with mutations identified in NAC responders. (C, D) Kaplan–Meier curves comparing overall survival and disease- or progression-free survival between wild-type and mutated *METTL3* in MIBC patients using the log-rank test. n, patient number.

We identified the somatic mutations of *CDH9*, *METTL3*, and *PTPRH* that were associated with NAC response, and *CCDC141* mutation that was associated with NAC resistance. In the subsequent investigation on the relationship between the mutations and prognosis, we compared the OS and disease-free survival (DFS) of BC patients who acquired wild-type or mutated *CDH9*, *METTL3* *PTPRH*, and *CCDC141* based on the data from the cBioPortal for Cancer Genomics (https://www.cbioportal.org/). Interestingly, MIBC patients bearing mutated *METTL3* had a significantly (*P*<0.05) longer OS and DFS as compared to the patients bearing wild-type *METTL3* (Figure 4A and B). However, MIBC patients harboring mutated *CDH9, PTPRH*, and *CCDC141* displayed similar OS or DFS as compared to the patients bearing the wild-type *CDH9, PTPRH* and *CCDC141*, respectively. Therefore, these data indicated that the somatic mutation of *METTL3* could be a good predictor of NAC response in MIBC patients.

We further analyzed the somatic mutations of *METTL3* and their effect on protein sequence. Herein, we identified two novel mutations of *METTL3*, one located in the methyltransferase domain (c. 1384 G>C, p. Q462E) while the other (c. 388 G>C, p. E130K) in the non-typical domain. A stick plot of METTL3 protein containing the amino acid alterations reported in BC samples and the new amino acid alterations identified in this study were displayed in Figure 4C. The methyltransferase domain of METTL3 revealed the locations of R529C, E532Q, P577R, E516K, Q462E, R468Q, and R471H in the three-dimensional space (Figure 4D). These results indicated that the somatic mutation of *METTL3* is a predictor of pathological response to NAC in BC patients.

## 4. Discussion

Administering chemotherapeutic drugs to the patients before surgical removal provides several advantages to cancer patients. For instance, NAC improves surgical resectability of tumor by reducing micrometastases, which are the trigger of metastasis. Moreover, cancer patients benefit from some advantages of NAC treatment from the aspects of drug resistance, pathological response, and survival rates [23]. At present, cisplatin-based NAC followed by radical cystectomy is the gold standard treatment for BC. Albeit its positive results in the treatment of BC, the 5-year overall survival rate of BC patients remains remaining low. Thus, whether this regimen is suitable for treating BC remains debatable [11]. Supported by some recent clinical trials and comparative analysis, BC patients receiving NAC had poor pathological response and no superior clinical outcomes [24,25].

The advance of NGS has shed the light on the genomic landscape of humans. Besides, information generated from NGS is beneficial to the development of precision oncology and personalized medicine [26]. For example, WES of breast cancer samples identified that the somatic mutation of *SIN3A* in breast cancer aggravated the tumor development [27]. Furthermore, WES of MIBC tumor samples revealed that somatic mutations of *UNC5C* and DNA repair genes contributed to prolonged survival [12,28]. In addition, the mutations of *ERCC2* [13] and *ERBB2* [29] were significantly enriched in responders. With the application of Sanger sequencing in our previous study, we showed that somatic mutation of *FGFR3* in MIBC patients is a potential predictive biomarker of NAC response [30]. This evidence suggests the potential of NGS in biomarker studies and personalized medicine development.

Since MIBC is a heterogeneous disease and exhibits inconsistent response to NAC, we utilized the WES in this study to investigate the potential biomarkers in predicting response to NAC in MIBC patients. In discovery cohort, the application of WES and bioinformatic analysis identified a list of mutated genes which could predict the pathological response to NAC. As the cause of cancer development, these genetic mutations are implicated in gene amplification, silencing, activation, and inactivation [31]. The somatic mutations of *CDH9*, *PTPRH*, and *METTL3* were exclusively altered in the NAC responders. These results indicate that these mutations could predict the response of BC patients receiving NAC.

Corroborated by the pathway enrichment analysis, these genes were involved in the regulation of adherens junctions and Hippo signaling pathway. As a typical cadherin, CDH9 mediates the cell-cell interactions and is only largely expressed in the late stage of epithelial-to-mesenchymal transition (EMT) [32]. These results suggest that the disruption of EMT regulated by CDH9 could predict the pathological response to NAC. However, the mutation of *CDH9* in BC patients receiving NAC was not found in the previous studies [12,13,28-30]. In this study, the mutations of *CDH9*, such as chr5:26885861 C>T and chr5: 26988395 A>C, were significantly enriched in NAC responders with a mutation frequency of 9/21.

Furthermore, the mutation of *PTPRH* was correlated with the regulation of adherens junctions in BC. Van Allen *et al*. reported that *PTPRH* mutations were present in 1/25 responders and 1/25 nonresponders, and there were no significant differences between the above two groups [13] (Table 2). Herein, *PTPRH* mutations, such as chr19: 55693222 G>T and chr19: 55693503 T>A, were found in 7/21 responders and 0/12 non-responders.

In addition, the dysregulation of RNA methyltransferase, *METTL3*, activated Hippo signaling pathway through the increased translation of Hippo pathway effector, TAZ [33]. Consequently, the dysregulation of Hippo pathway triggered migration and metastatic properties of cancer cells [33]. In the study of Van Allen *et al.*, *METTL3* mutations were found in 2/25 responders and 0/25 non-responders, and there were no significant differences between these two groups [13] (Table 2). Herein, *METTL3* mutations were detected in 8/21 responders and 0/12 non-responders, in which 5/8 responders acquired c. 1384 G>C mutation and 3/8 responders acquired c. 388 G>C mutation.

Plimack *et al*. found that *ATM*, *RB1*, and *FANCC* were highly mutated in NAC responders [12]. In this study, *ATM* mutations were found in 2/5 responders and 0/8 non-responders (*P*=0.05), *RB1* mutations in 1/5 responders and 2/8 non-responders (*P*=0.83), and *FANCC* mutations in 0/5 responders and 1/8 non-responders (*P*=0.41). In addition, the mutations of *ERCC2* [13] and *ERBB2* [29] were significantly enriched in responders. However, in this study, *ERBB2* mutations were found in 0/5 responders and 1/8 non-responders (*P*=0.41), and *ERCC2* mutations in 1/5 responders and 1/8 non-responders (*P*=0.72). Our previous study identified that the somatic mutation of *FGFR3* in MIBC patients is a potential biomarker in predicting the NAC response [30]. However, in the present study, *FGFR3* was found to be mutated in 0/5 responders and 1/8 non-responders (*P*=0.41).

In contrast, the somatic mutation of *CCDC141* was associated with the NAC nonresponders, indicating that *CCDC141* mutation is responsible for the resistance of NAC in BC patients. Van Allen *et al*. reported that *CCDC141* mutations were present in 0/25 responders and 1/25 non-responders and there were no significant differences between these two groups [13] (Table 2). Herein, *CCDC141* mutations, such as chr2: 179839888 G>C, chr2: 179698970 C>G, and chr2: 179733841 T>C, were detected in 0/21 responders and 5/12 non-responders. The differences in races, treatment methods, and sample sizes in different studies may account for the discrepancies of above-mentioned results. Therefore, further experiments should be carried out to validate the findings in larger cohorts.

Further survival studies demonstrated that the BC patients acquiring mutated *METTL3* had the most significant survival benefits after NAC treatment as compared to the patients acquiring wild-type *METTL3*. This prompted us to further discuss the role of *METTL3* in predicting the NAC response in cancer patients. Biologically, METTL3 and its cofactors make up the m6A methyltransferase complex (MTC) that catalyzes RNA methylation, which is a vital process in determining the cell fate, especially in endothelial-to-hematopoietic transition during embryogenesis [34]. In support of our findings, the upregulation of METTL3 expression promotes BC development through AFF4/NF-kb signaling pathway, and subsequently represses the expression of tumor suppressor gene PTEN [35]. Furthermore, high METTL3 and YAP activities restrict the reduction of cell proliferation on drug treatment in NSCLC, indicating the potential of *METTL3* dysregulation in conferring drug resistance in BC [36]. With these in mind, the somatic mutation of *METTL3* can be a potential candidate in predicting the pathological response to NAC in MIBC patients. Due to the small number of samples used in this study, the diagnostic potential of *METTL3* should be further validated in larger cohorts.

## 5. Conclusion

Our findings illustrated that the somatic mutation of *METTL3* could predict the pathological response to NAC in MIBC patients. With more in-depth elucidation of its molecular mechanisms, the mutation could be an ideal biomarker for diagnostic purposes and could assist in the development of a novel targeted therapy for BC in future.

## References

[1] Richters A, Aben KK, Kiemeney L (2019). The Global Burden of Urinary Bladder Cancer:An Update. World J Urol.

[2] Bray F, Ferlay J, Soerjomataram I, Siegel RL, Torre LA, Jemal A (2018). Global Cancer Statistics 2018:GLOBOCAN Estimates of Incidence and Mortality Worldwide for 36 Cancers in 185 Countries. CA Cancer J Clin.

[3] Czerniak B, Dinney C, McConkey D (2016). Origins of Bladder Cancer. Annu Rev Pathol.

[4] Kassouf W, Traboulsi SL, Kulkarni GS, Breau RH, Zlotta A, Fairey A (2015). CUA guidelines on the Management of Non-muscle Invasive Bladder Cancer. Can Urol Assoc J.

[5] Cooley LF, McLaughlin KA, Meeks JJ (2020). Genomic and Therapeutic Landscape of Non-muscle-invasive Bladder Cancer. Urol Clin North Am.

[6] Ghandour R, Singla N, Lotan Y (2019). Treatment Options and Outcomes in Nonmetastatic Muscle Invasive Bladder Cancer. Trends Cancer.

[7] Kamoun A, de Reyniès A, Allory Y, Sjödahl G, Robertson AG, Seiler R (2019). A Consensus Molecular Classification of Muscle-invasive Bladder Cancer. Eur Urol.

[8] Choi W, Porten S, Kim S, Willis D, Plimack ER, Hoffman-Censits J (2014). Identification of Distinct Basal and Luminal Subtypes of Muscle-Invasive Bladder Cancer with Different Sensitivities to Frontline Chemotherapy. Cancer Cell.

[9] Sherif A, Holmberg L, Rintala E, Mestad O, Nilsson J, Nilsson S (2004). Neoadjuvant Cisplatinum Based Combination Chemotherapy in Patients with Invasive Bladder Cancer:A Combined Analysis of Two Nordic Studies. Eur Urol.

[10] Advanced Bladder Cancer Meta-Analysis Collaboration. Neoadjuvant Chemotherapy in Invasive Bladder Cancer:Update of a Systematic Review and Meta-analysis of Individual Patient Data Advanced Bladder Cancer (ABC) Meta-analysis Collaboration (2005). Eur Urol.

[11] Gakis G (2020). Management of Muscle-invasive Bladder Cancer in the 2020s:Challenges and Perspectives. Eur Urol Focus.

[12] Plimack ER, Dunbrack RL, Brennan TA, Andrake MD, Zhou Y, Serebriiskii IG (2015). Defects in DNA Repair Genes Predict Response to Neoadjuvant Cisplatin-based Chemotherapy in Muscle-invasive Bladder Cancer. Eur Urol.

[13] Van Allen EM, Mouw KW, Kim P, Iyer G, Wagle N, Al-Ahmadie H (2014). Somatic ERCC2 Mutations Correlate with Cisplatin Sensitivity in Muscle-invasive Urothelial Carcinoma. Cancer Discov.

[14] Berger MF, Mardis ER (2018). The Emerging Clinical Relevance of Genomics in Cancer Medicine. Nat Rev Clin Oncol.

[15] Beltran H, Eng K, Mosquera JM, Sigaras A, Romanel A, Rennert H (2015). Whole-Exome Sequencing of Metastatic Cancer and Biomarkers of Treatment Response. JAMA Oncol.

[16] Miyamoto DT, Mouw KW, Feng FY, Shipley WU, Efstathiou JA (2018). Molecular Biomarkers in Bladder Preservation Therapy for Muscle-Invasive Bladder Cancer. Lancet Oncol.

[17] Chen R, Im H, Snyder M (2015). Whole-Exome Enrichment with the Agilent SureSelect Human All Exon Platform. Cold Spring Harb Protoc.

[18] Wang K, Li M, Hakonarson H (2010). ANNOVAR:Functional Annotation of Genetic Variants from High-throughput Sequencing Data. Nucleic Acids Res.

[19] Cibulskis K, Lawrence MS, Carter SL, Sivachenko A, Jaffe D, Sougnez C (2013). Sensitive Detection of Somatic Point Mutations in Impure and Heterogeneous Cancer Samples. Nat Biotechnol.

[20] Saunders CT, Wong WS, Swamy S, Becq J, Murray LJ, Cheetham RK (2012). Strelka:Accurate Somatic Small-variant Calling from Sequenced Tumor-normal Sample Pairs. Bioinformatics.

[21] Nik-Zainal S, Alexandrov LB, Wedge DC, Van Loo P, Greenman CD, Raine K (2012). Mutational Processes Molding the Genomes of 21 Breast Cancers. Cell.

[22] Robertson AG, Kim J2, Al-Ahmadie H, Bellmunt J, Guo G, Cherniack AD (2017). Comprehensive Molecular Characterization of Muscle-Invasive Bladder Cancer. Cell.

[23] Nguyen DP, Thalmann GN (2017). Contemporary Update on Neoadjuvant Therapy for Bladder Cancer. Nat Rev Urol.

[24] Schinzari G, Monterisi S, Pierconti F, Nazzicone G, Marandino L, Orlandi A (2017). Neoadjuvant Chemotherapy for Patients with Muscle-invasive Urothelial Bladder Cancer Candidates for Curative Surgery:A Prospective Clinical Trial Based on Cisplatin Feasibility. Anticancer Res.

[25] Hanna N, Trinh QD, Seisen T, Vetterlein MW, Sammon J, Preston MA (2018). Effectiveness of Neoadjuvant Chemotherapy for Muscle-invasive Bladder Cancer in the Current Real World Setting in the USA. Eur Urol Oncol.

[26] Gagan J, Van Allen EM (2015). Next-generation Sequencing to Guide Cancer Therapy. Genome Med.

[27] Watanabe K, Yamamoto S, Sakaguti S, Isayama K, Oka M, Nagano H (2018). A Novel Somatic Mutation of SIN3A Detected in Breast Cancer by Whole-exome Sequencing Enhances Cell Proliferation through ERalpha Expression. Sci Rep.

[28] Yap KL, Kiyotani K, Tamura K, Antic T, Jang M, Montoya M (2014). Whole-exome Sequencing of Muscle-invasive Bladder Cancer Identifies Recurrent Mutations of UNC5C and Prognostic Importance of DNA Repair Gene Mutations on Survival. Clin Cancer Res.

[29] Groenendijk FH, de Jong J, van de Putte EE, Michaut M, Schlicker A, Peters D (2016). ERBB2 Mutations Characterize a Subgroup of Muscle-invasive Bladder Cancers with Excellent Response to Neoadjuvant Chemotherapy. Eur Urol.

[30] Yang Z, Zhang R, Ge Y, Qin X, Kang X, Wang Y (2018). Somatic FGFR3 Mutations Distinguish a Subgroup of Muscle-Invasive Bladder Cancers with Response to Neoadjuvant Chemotherapy. EBioMedicine.

[31] Du Y, Grandis JR (2015). Receptor-type Protein Tyrosine Phosphatases in Cancer. Chin J Cancer.

[32] Thedieck C, Kalbacher H, Kuczyk M, Muller GA, Muller CA, Klein G (2007). Cadherin-9 is a Novel Cell Surface Marker for the Heterogeneous Pool of Renal Fibroblasts. PLoS One.

[33] Han Y (2019). Analysis of the Role of the Hippo Pathway in Cancer. J Transl Med.

[34] Deng X, Su R, Weng H, Huang H, Li Z, Chen J (2018). RNA N^6^-methyladenosine Modification in Cancers:Current Status and Perspectives. Cell Res.

[35] Han J, Wang JZ, Yang X, Yu H, Zhou R, Lu HC (2019). METTL3 Promote Tumor Proliferation of Bladder Cancer by Accelerating Pri-miR221/222 Maturation in m6A-dependent Manner. Mol Cancer.

[36] Jin D, Guo J, Wu Y, Du J, Yang L, Wang X (2019). m^6^A mRNA Methylation Initiated by METTL3 Directly Promotes YAP Translation and Increases YAP Activity by Regulating the MALAT1-miR-1914-3p-YAP Axis to Induce NSCLC Drug Resistance and Metastasis. J Hematol Oncol.

